# Acetylsalicylic Acid Promotes Corneal Epithelium Migration by Regulating Neutrophil Extracellular Traps in Alkali Burn

**DOI:** 10.3389/fimmu.2020.551057

**Published:** 2020-10-15

**Authors:** Ting Wan, Yue Zhang, Kelan Yuan, Jinjin Min, Yujie Mou, Xiuming Jin

**Affiliations:** Eye Center, Second Affiliated Hospital, School of Medicine, Zhejiang University, Hangzhou, China

**Keywords:** migration, corneal epithelium, alkali burn, neutrophil extracellular traps, acetylsalicylic acid (ASA)

## Abstract

Neutrophils are the first cells to migrate into the cornea in response to alkali burns, and excessive neutrophil infiltration is associated with inflammatory injury and a poorer prognosis. In an effort to understand the mechanisms underlying the inflammation mediated by neutrophils after alkali burns, we examined the role of alkali-activated neutrophils on human corneal epithelial cells (HCEs) proliferation and migration, as well as the effects of acetylsalicylic acid (ASA) and dexamethasone (DXM) on NETosis. We stimulated human neutrophils with sodium hydroxide (NaOH) and observed dose- and time-dependent neutrophil extracellular traps (NETs) formation. We also observed that ASA, but not DXM, significantly inhibited NaOH-induced NETosis. Furthermore, the activation of nuclear factor (NF)-κB, but not the production of reactive oxygen species, was involved in ASA-regulated NETosis. Moreover, NETs were found to be involved in alkali-activated neutrophils (ANs) induced neutrophil-HCE adhesion. ANs enhanced HCEs proliferation via phagocytosis. Meanwhile, ANs inhibited HCEs migration through the release of NETs, which was partially rescued by 5 mM ASA. In conclusion, ANs may interfere with HCEs proliferation and migration by phagocytosis and NETs formation, respectively. ASA may enhance HCEs migration by decreasing NETs formation through inhibition of NF-κB activation and could be a promising strategy for improving the prognosis of corneal alkali burns.

## Introduction

Ocular alkali burns, which account for 12–22% of ocular injuries (1), are vision-threatening emergencies that require prompt management. They rarely heal spontaneously and could lead to recurrent epithelium erosion, ulceration, neovascularization, extensive formation of scar tissue, and consequent visual impairment (2). Although keratoplasty is a feasible therapy for treating corneal scars in the chronic phase, the outcome of this surgery is largely dependent on whether the destructive responses post alkali injury are controlled in the early stages. Thus, appropriate treatment during the early period after an alkali burn is important for promoting corneal healing and decreasing corneal opacity. To date, the precise mechanisms of alkali-induced destruction remain elusive, and there is still a lack of proper and effective clinical treatments.

Once an alkali burn occurs, immune cells are mobilized to the injured tissues and are accompanied by rises in pro-inflammatory cytokine levels (3, 4). Among these cells, neutrophils are the first to migrate into the tissues in response to insults and are reported to be highly involved in the pathophysiological progression of corneal alkali burns. Neutrophils not only remove tissue debris resulting from an alkali burn but are also involved in the initiation of cellular proliferation and scar formation through releasing growth factors and pro-angiogenic factors (5, 6). Moreover, neutrophils are known to exert negative influences on wound healing in corneal alkali burn models. Extensive infiltration of neutrophils has been observed in corneal tissues after alkali burns and is associated with poor outcomes. Previously, neutrophils have been demonstrated to engulf and degrade the pathogens intracellularly, while also releasing microbicidal proteins and reactive oxygen species (ROS) (7). More recently, another effector mechanism of neutrophils has been discovered in which they release neutrophil extracellular traps (NETs), which exacerbate inflammation and tissue damage. NETs are characterized as net-like structures containing not only extracellular DNA and histones, but also cytoplasmic proteases and antimicrobial peptides, as well as oxidant molecules (8). Excessive NETs formation has been reported to be involved in the development of transfusion-related acute lung injury, autoimmune diseases, and thrombosis (9–11). Furthermore, excessive NETs formation can also contribute to ocular inflammatory diseases, such as dry-eye disease and diabetic retinopathy (12, 13). However, little is known about the mechanisms underlying neutrophil-mediated inflammatory responses to alkali burns. New approaches involving the manipulation of neutrophil-related inflammatory injury may shed light on the medical treatment of corneal alkali burns.

Rapid epithelial wound healing, a key element of successful corneal rehabilitation, is a complicated process requiring the coordination of cells, growth factors, and cytokines (14, 15). A failure in re-epithelialization could lead to a higher incidence of infection, increased severity of inflammation, and dysfunctional stromal remodeling, which in turn result in corneal scarring and visual loss. Therefore, in the present study, we investigated the involvement of NETs in alkali burns, and elucidated the effect of alkali-activated neutrophils (ANs) on corneal epithelial cell proliferation and migration. Moreover, as NETs are highly correlated with inflammation, we also assessed the effects of commonly used anti-inflammatory drugs, including acetylsalicylic acid (ASA) and dexamethasone (DXM), on NETs formation and epithelial cell function.

## Methods

### Reagents

Percoll was purchased from GE Healthcare (Little Chalfont, UK). Phorbol 12-myristate 13-acetate (PMA), DNase I, Sodium hydroxide (NaOH), cytochalasin D, ASA, DXM, Calcein AM, and dichlorofluorescein diacetate (DCF-DA) were purchased from Sigma-Aldrich (St. Louis, MO, USA). Anti-histone H3 antibody, anti-phosphorylated nuclear factor κB (anti-p-NF-κB, p65) antibody, horseradish peroxidase (HRP) secondary antibody, and secondary antibodies coupled to AF488 or AF555 were purchased from Santa Cruz Biotechnology (CA, USA). Anti-myeloperoxidase (MPO) antibody and anti-β actin antibody were purchased from Abcam(Cambridge, UK). SYTOX Green, Quant-iTPicoGreen double-stranded deoxyribonucleic acid assay kits were purchased from ThermoFisher Scientific (Basingstoke, UK). Cell proliferation reagent WST-1 was purchased from Roche (Basal, Switzerland).

### Isolation of Human Neutrophils

The present study was conducted with adherent to the tenets of the Declaration of Helsinki, and was approved by the ethics committee of Second Affiliated Hospital, School of Medicine, Zhejiang University, China. All participants participating in the investigation provided written informed consent for the collection and subsequent analyses of blood. Neutrophils were isolated from the peripheral blood of fasting healthy donors has been described before using Percoll gradient centrifugation, (16). For each donor, 10 to 30 ml blood was drawn, and bloods from at least three donors were used to repeat the same assay. The isolated cells contained >96% neutrophils as determined by Wright–Giemsa staining, with 98% cell viable as determined by Trypan blue staining. The isolated cells (4 × 10^5^/ml) were finally cultured in RPMI 1,640 medium supplied with 2% bovine serum albumin.

### Neutrophil Stimulation

Neutrophils (2 × 10^5^ cells in 500 μL of media per well) were treated with PMA (50 nM) or NaOH of specific concentrations and placed in a humidified incubator at 37°C with 5% CO2 for a designated period. In some assays, neutrophils were first incubated with ASA (1 or 5 mM), DXM (10 or 50 μM), or vehicle (controls) for 30 min. In one experiment, stimulated neutrophils were treated with DNase I (100 U/mL) to degrade the structure of NETs as a control. All chemicals were freshly prepared before use.

### Identification and Quantification of NETs Formation

For indentifying the structure of NETs, stimulated neutrophils were incubated with primary anti-MPO and anti-histone H3 antibodies after fixation and blocking, and then stained with secondary antibodies coupled to AF555 or AF488. Meanwhile, DNA was detected by 4′, 6′-diamidino-2-phenylindole (DAPI) staining. After mounting, the NETs structures were observed and analyzed using confocal microscope (Olympus IX-50). NETs were also accessed by adding a membrane-impermeable DNA-binding dye SYTOX green (5 μM, Molecular Probes, Invitrogen Life Technologies) to the culture medium after specific stimulation. SYTOX green stained dead-cell cultures were imaged with an inverted fluorescence microscope (Olympus IX-50). Besides, quantification of the extracellular DNA in supernatants was accomplished by using Quant-iT PicoGreen dsDNA assay kit according to the manufacturer's instructions, as previously described (17).

### ROS Production

Neutrophils were incubated in Ca^2+^ and Mg^2+^ free PBS with 10 μM DCF-DA at 37°C for 20 min, and then transferred to a 96-well plate. Subsequently, the neutrophils (1 × 10^6^ cells/well) were pretreated with vehicle or ASA for 30 min, and then stimulated with 5 mM NaOH for 30 min. The fluorescence was accessed using SpectraMax M3 fluorescent plate reader with the excitation wavelength as 480 nm and the emission wavelength as 520 nm.

### Immunoblotting

The neutrophils (3 × 10^6^ cells/well) were pretreated with vehicle, ASA or DXM for 30 min and then stimulated with NaOH (5 mM) for another 30 min. After preparing cells lysates with 1× loading buffer, equal amounts of boiled proteins from each sample were run on 12% sodium dodecyl sulfate-polyacrylamide gel followed by electrotransferring onto polyvinylidenefluoride membranes. After blocking with 5% bovine serum albumin, membranes were incubated with anti- phospho-NF-κBp65 and anti-β actin antibody overnight at 4°C, and then with HRP-conjugated secondary antibody for 2 h at room temperature. Protein bands were visualized by enhanced chemiluminescence. The gray degree of protein bands was detected by image J, and the value of p-NF-κB p65/β actin was calculated.

### Cell Culture Experiments

Human corneal epithelial cells (HCEs) were purchased from American Type Culture Collections. The cells (3 × 10^5^/mL) were cultured in Dulbecco's Modified Eagle Medium–Nutrient Mixture F12 (DMEM/F12, Gibco) supplemented with bovine serum albumin (10%) and treated with different stimuli as described in the following assays.

To investigate the effect of alkali-activated neutrophils (ANs) on epithelial cells, neutrophils were activated with NaOH (5 mM), after which the culture medium was neutralized using hydrochloric acid. For experiments investigating the effect of alkali together with ANs, the culture medium was not neutralized, and the final NaOH concentration was 0.5 mM. DNase I (100 U/mL), which degrades the structure of NETs, was used to assess the function of neutrophil phagocytosis after alkali stimulation. Cytochalasin D (100 μg/mL), which inhibits the ability of neutrophils to perform phagocytosis, was used to examine the function of NETs after alkali stimulation.

### Neutrophil-HCE Adhesion Assay

HCEs (5.0 × 10^4^ cells/well) were cultured and allowed to adhere overnight in 12-well plates (Costar). Meanwhile, neutrophils (5 × 10^5^ cells/mL) were labeled with Calcein AM. After washing in warm phenol red-free DMEM/F12, neutrophils were co-cultured with HCEs for 2 h in the presence of a vehicle, 0.5 mM NaOH, ANs, or ANs with DNase I. Subsequently, non-adherent neutrophils were gently removed by washing with DMEM/F12. The adherent neutrophils were imaged using an inverted fluorescence microscope (Olympus IX-50) and quantified using Image J. Adhesion was quantified by calculating the ratio of the number of neutrophils to that of HCEs.

### Assessment of HCE Proliferation

The proliferation of HCEs was examined by performing the WST-1 based colorimetric assay according to the manufacturer's instructions. Briefly, the HCEs were plated into 96-well plates at densities of 2,500, 5,000, 10,000, and 20,000 cells/mL and incubated overnight. The HCEs were then co-cultured with vehicle, ANs, ANs with DNase I, ANs with cytochalasin D, ANs with ASA, or ANs with DXM. After 24 h of incubation, 10 μL of WST-1 was added and the cells were incubated for another 4 h. The proliferation of HCEs was quantified by measuring the absorbances of the wells at the 450 and 630 nm wavelengths.

### HCE Migration Assay

The migration of HCEs was examined by performing the scratch-wound assay on confluent monolayer of HCEs. Briefly, HCEs (5 × 10^4^ cells/well) were seeded into 12-well plates and allowed to reach confluence within 24 h. Then, a 200 μL pipette tip was used to scrape the confluent monolayer of HCEs, and the cell debris was removed by gentle washing with phosphate-buffered saline (PBS). Afterwards, the HCEs were incubated with vehicle, 0.5 mM NaOH, ANs, ANs with DNase I, ANs with cytochalasin D, ANs with ASA, or ANs with DXM in growth media. Images were taken after 24 h using a BH-2 Multifunctional Optical Microscope (Olympus) after crystal violet staining. Cell migration was quantified by assessing the wound closure percentage across the scratch, as calculated using the following formula:

Wound closure %=W0–Wn/W0, where Wn is the width of the gap 24 h after scratching, and W0 is the initial width of the gap immediately after scratching.

### Statistical Analysis

All results were presented as mean ± Standard deviation (SD). Statistical analyses were performed using the Statistical Package for Social Sciences, version 13. The Student's *t*-test was used to compare normally distributed data of two groups. Comparisons between multiple groups were performed using one-way analysis of variance (ANOVA). Results were considered statistically significant when *p* < 0.05.

## Results

### Alkali Conditions Induce Dose- and Time-Dependent NETs Formation

**Figure 1:**
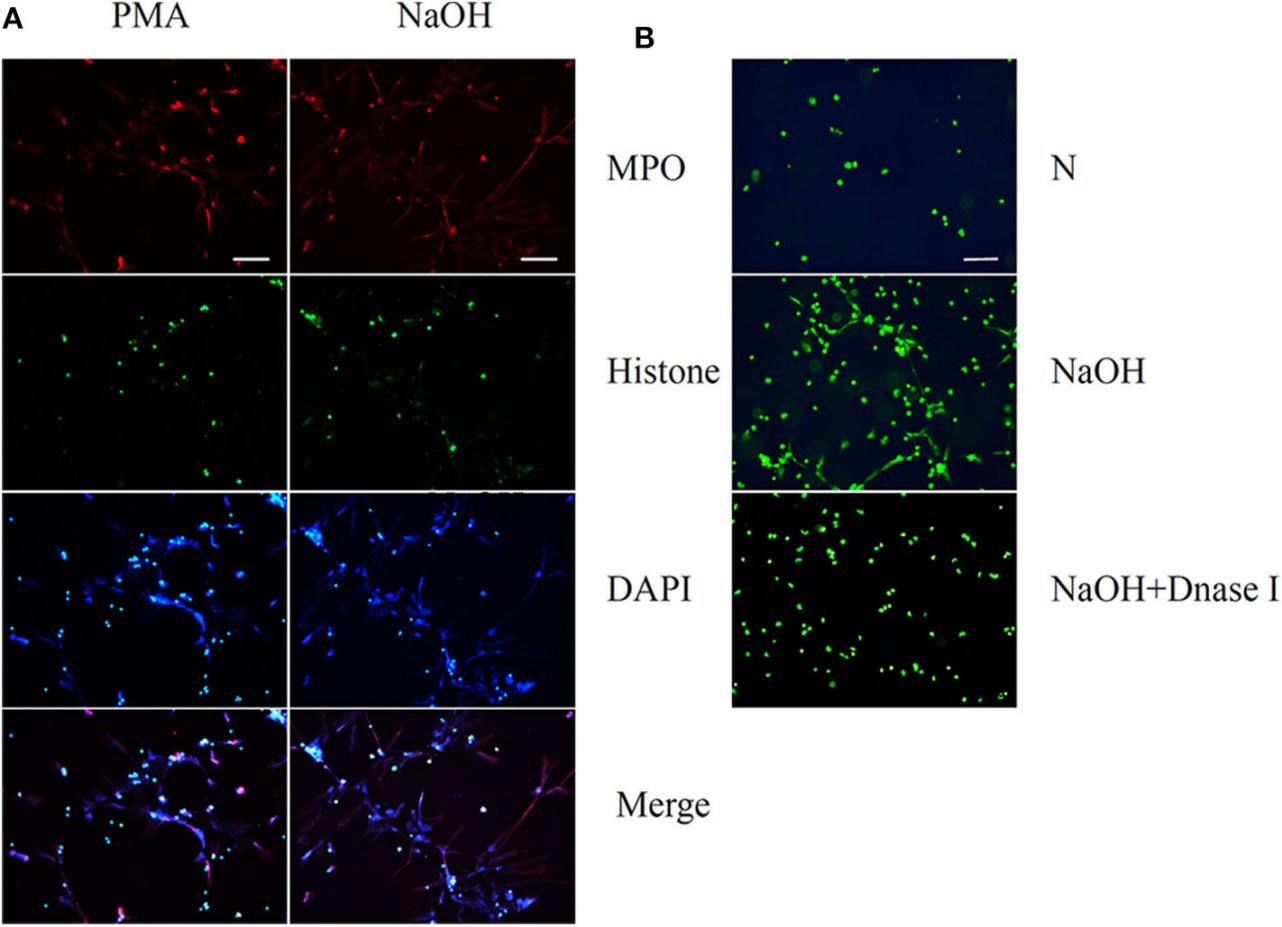
Phorbol 12-myristate 13-acetate (PMA) and Sodium hydroxide (NaOH) stimulate neutrophil extracellular traps (NETs) formation in human neutrophils. **(A)** Human neutrophil suspended in media were treated with PMA (50 nM) for 2 h or NaOH (5 mM) for 30 min. Neutrophils were labeled with 4′, 6′-diamidino-2-phenylindole (DAPI) to identify DNA (blue) and with antibodies to identify neutrophil histone (green) and MPO (red). **(B)** NaOH stimulated NETs formation could be degraded by DNase I as observed by SYTOX green staining. Bar: 75 μm.

**Figure 2:**
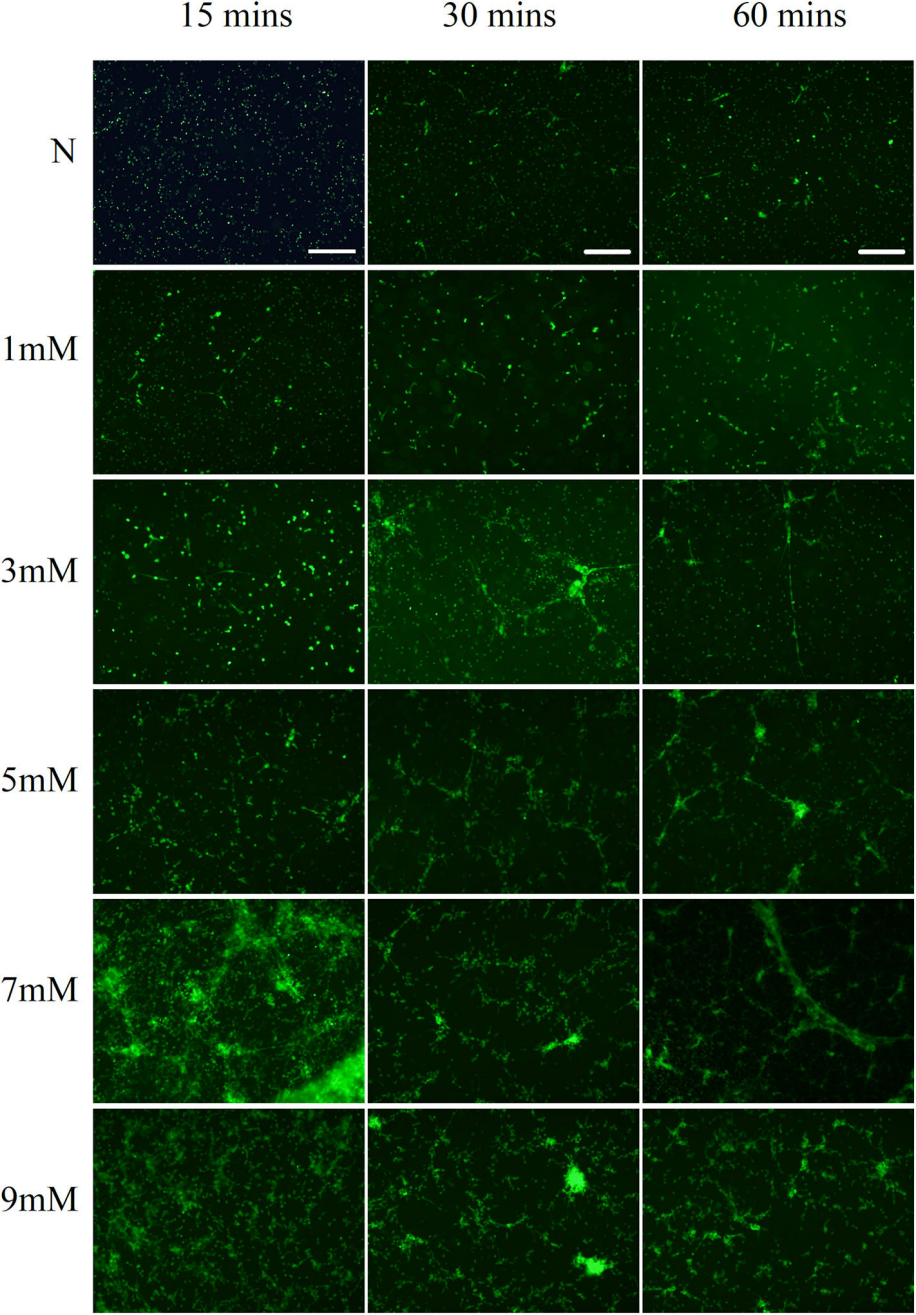
Alkali induces dose- and time-dependent NETs formation. Alkali triggered dose- and time-dependent NETs formation as demonstrated by SYTOX green staining. While only a few NETs structures were observed 60 min after 1 mM NaOH and 3 mM NaOH treatment, 5 mM NaOH stimulation for 30 min induced significant NETs generation. Moreover, many neutrophils died and detached as early as 15 min after both 7 mM NaOH and 9 mM NaOH treatment. Bar: 250 μm.

The neutrophils exhibited NETs structures as observed by microscopy after PMA (50 nM) stimulation for 2 h and NaOH (5 mM) stimulation for 30 min (Figure 1A). Neutrophils were labeled with antibodies to identify myeloperoxidase (MPO, red) and histones (green), while DNA (blue) was labeled with 4′,6-diamidino-2-phenylindole (DAPI). Immunofluorescence staining confirmed the presence of PMA- and alkali-induced NETs formation. Furthermore, NaOH (5 mM) stimulated NETs formation could be degraded by DNase I as observed by SYTOX green staining (Figure 1B). Additionally, the time- and dose-dependent formation of NETs triggered by alkali is shown in Figure 2 with SYTOX green staining. While only a few NETs structures were observed 60 min after 1 mM NaOH and 3 mM NaOH treatment, 5 mM NaOH stimulation for 30 min induced significant NETs generation. Moreover, many neutrophils died and detached as early as 15 min after both 7 mM NaOH and 9 mM NaOH treatment. From the above results, a 5 mM NaOH concentration and a stimulation time of 30 min were chosen for subsequent experiments.

### Alkali-Induced NETs Formation Is Inhibited by ASA, but Not by DXM

**Figure 3:**
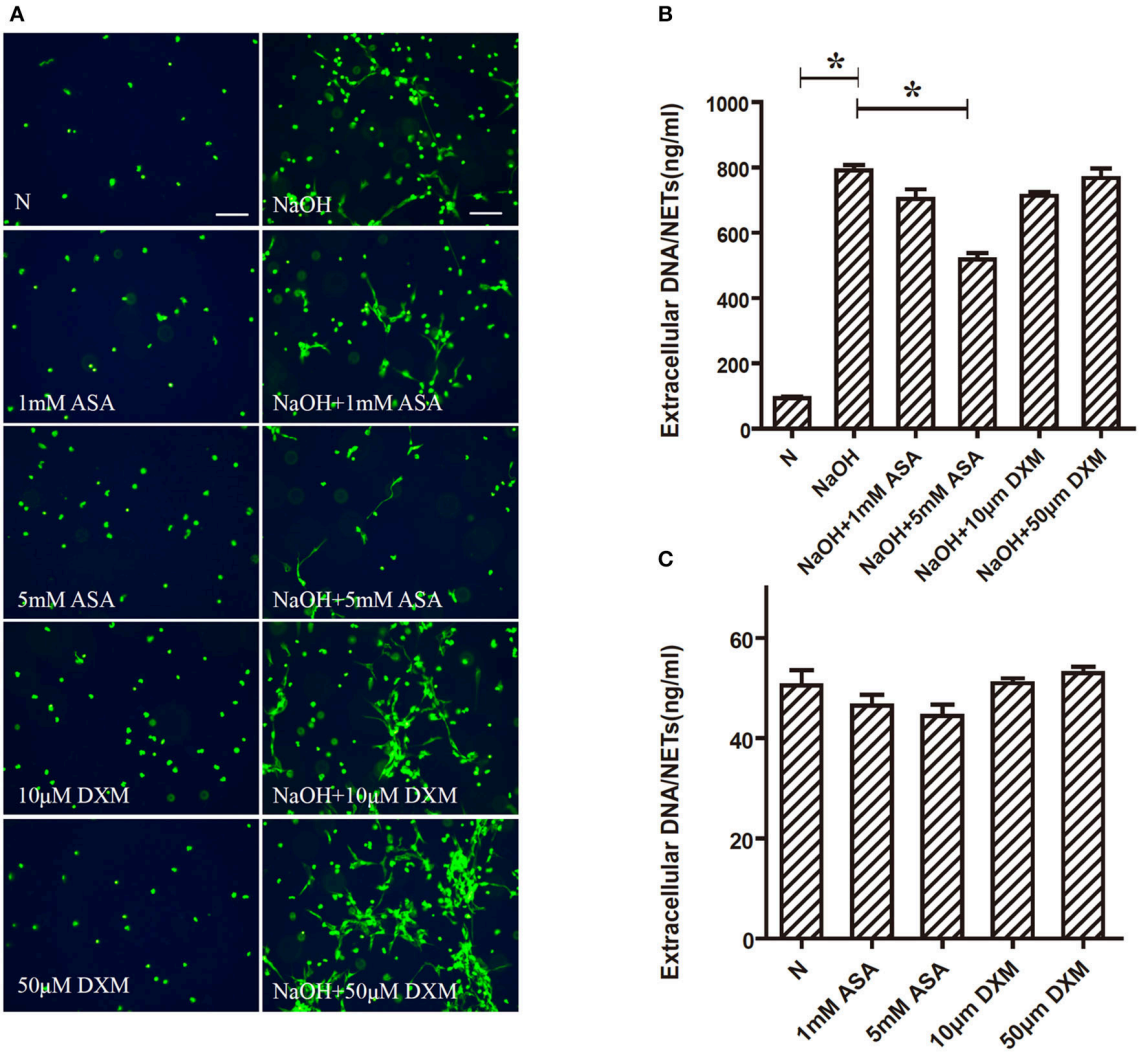
Alkali induced NETs formation is inhibited by ASA, but not by DXM. Human neutrophils suspended in media were pretreated for 30 min with ASA, DXM, or vehicle, and then NETs formation 30 min after stimulation with NaOH (5 mM) was examined using the membrane-impermeable DNA-binding dye SYTOX green and quantified by Quant-iTPicoGreen double-stranded deoxyribonucleic acid assay kit. **(A)** 5 mM ASA significantly inhibited alkali induced NETs formation. DXM and 1 mM ASA did not have any effect **(B)** Quantification of extracellular DNA confirmed that NaOH induced NETs formation was inhibited by ASA, but not by DXM. **(C)** Neither ASA nor DXM could induce NETs formation. The assay was repeated for three times with bloods from three different donors; error bars represent SD. *p < 0.05. Bar: 75 μm.

Fluorescence microscopy revealed that 5 mM ASA significantly inhibited alkali–induced NETs formation. However, DXM and 1 mM ASA did not have any effect (Figure 3A). To further verify the effects of ASA and DXM on alkali-induced NETosis, NETs formation was quantified by measuring the extracellular DNA in the supernatants. This assay corroborated that alkali-stimulated NETs formation was significantly decreased by 5 mM ASA (*p* < 0.05). In contrast, both DXM-treated and 1 mM ASA-treated neutrophils exhibited similar amounts of alkali-induced NETs formation when compared to the controls (*p* > 0.05) (Figure 3B).

### NF-κB Activation, but Not ROS Production, Is Involved in ASA-Regulated NETs Formation

**Figure 4:**
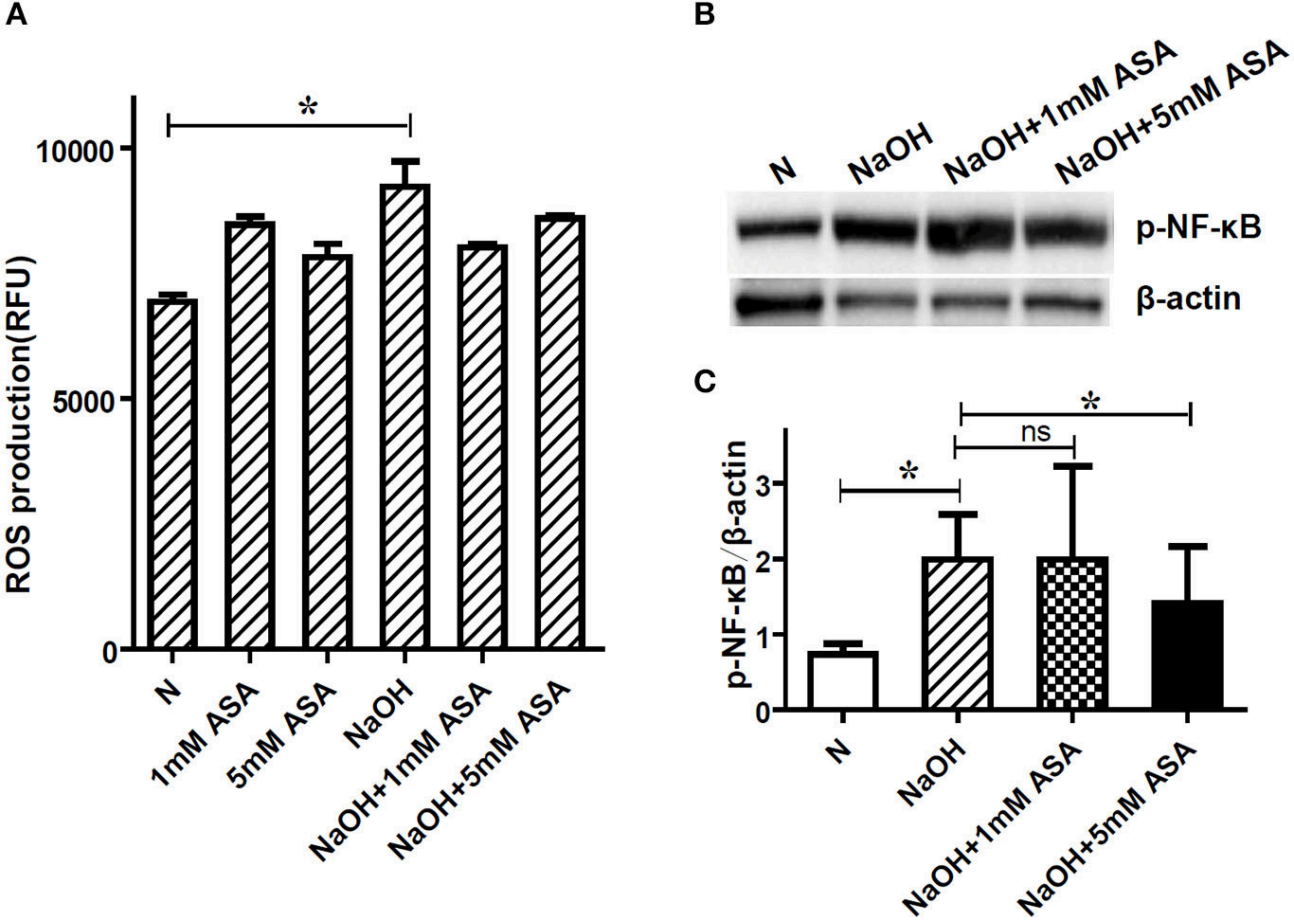
NF-κB activation, but not ROS production is involved in ASA regulated NETs formation. Neutrophils were pretreated for 30 min with ASA, DXM, or vehicle, and then stimulated with NaOH (5 mM) for 30 min. **(A)** ROS production was determined by dichlorofluorescein diacetate fluorescence. NaOH elicited significant neutrophil oxidative burst, but ASA treatment neither increased nor decreased this response. **(B)** NF-κB activation after alkali stimulation was determined by Western blot. **(C)** The gray degree of protein bands was detected by image J, and the value of p-NF-κB p65/β-actin was calculated. Quantification showed that p-NF-κB (p65) expression was significantly higher when the cells were stimulated with NaOH. This effect was inhibited by 5 mM ASA, but not modified by 1 mM ASA. Data represent mean ± SD of triplicate experiments, *p < 0.05.

Previous reports have thoroughly demonstrated that NETs could be formed via ROS-dependent or -independent pathways (18). In the present study, ROS generation was examined using a DCF-DA fluorescence assay to determine its role in alkali–induced NETs formation. As expected, there was a significant oxidative burst in neutrophils upon alkali induction (*p* < 0.05). Furthermore, ROS production by alkali-activated neutrophils was also examined with ASA pre-treatment to determine if ASA-regulated NETs formation is ROS-dependent. Our results showed that neither 1 mM ASA nor 5 mM ASA had a significant effect on alkali-induced ROS production (*p* > 0.05; Figure 4A).

The phosphorylation of the transcription factor NF-κB, which is a critical element in the inflammatory environment, has been previously reported to contribute to NETs formation (19). To this end, we also examined the effect of ASA on alkali-induced NF-κB phosphorylation. The expression of p-NF-κB (p65) in neutrophils was obviously elevated after alkali stimulation, and this effect was inhibited by 5 mM ASA (Figures 4B,C, Supplementary Figure 1). Conversely, 1 mM ASA did not affect alkali–induced NF-κB activation.

### NETs Are Involved in AN-Induced Neutrophil-HCE Adhesion

**Figure 5:**
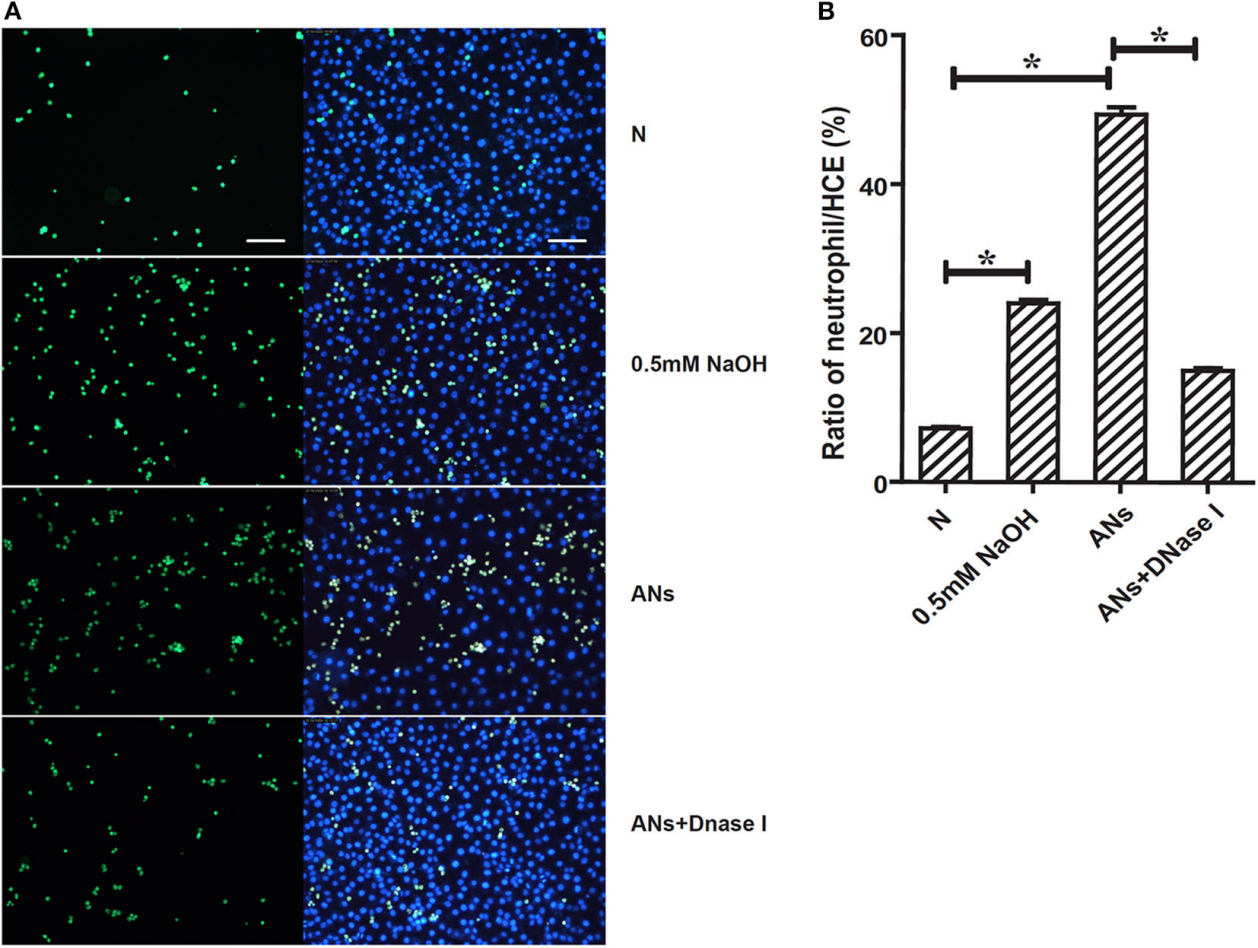
NETs are involved in AN-induced neutrophil-HCE adhesion. **(A)** Images presented showed the adhesion of Calcein AM (green) stained live neutrophils to corneal epithelial cells exposed to vehicle, 0.5 mM NaOH, AN, or AN with DNase I. **(B)** Quantification of the mean ratio of neutrophil/HCE showed that ANs significantly increased the neutrophil-HCE adhesion as compared with both control cells and cells treated with 0.5 mM NaOH. Moreover, this response could be significantly inhibited by DNase I. Data represent mean ± SD of triplicate experiments, *p < 0.05. Bar: 75 μm.

As shown in Figure 5, 0.5 mM NaOH enhanced the adhesion of neutrophils to the surface of HCEs as compared to cells cultured with normal DMEM/F12 (*p* < 0.05). Meanwhile, ANs significantly increased the neutrophil-HCE adhesion ratios compared to both control cells and cells treated with 0.5 mM NaOH (*p* < 0.05). Moreover, this AN-induced increase in neutrophil-HCE adhesion was significantly inhibited by DNase I (*p* < 0.05), which eliminates NETs by degradation. This suggests that NETs are involved in AN-induced neutrophil-HCE adhesion.

### ANs Promote HCE Proliferation via Phagocytosis, Which Could Be Inhibited by 5 mM ASA

**Figure 6:**
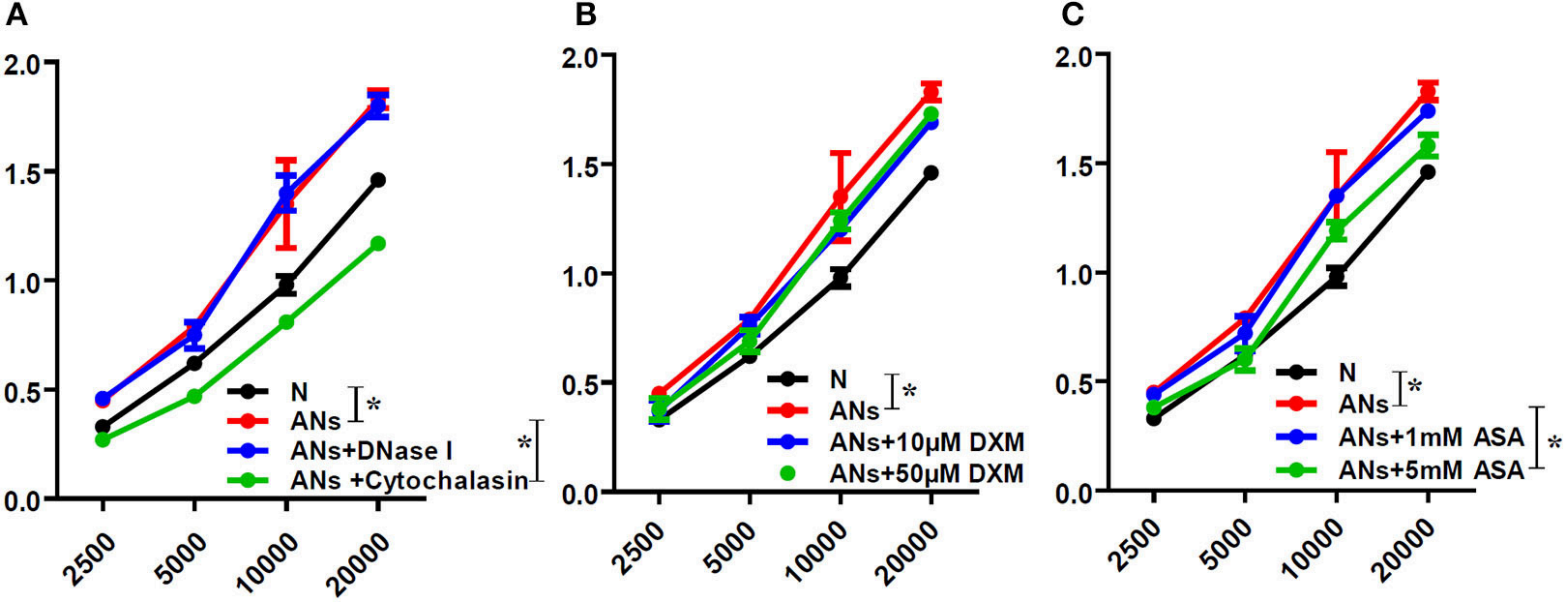
ANs promote HCE proliferation via phagocytosis, which could be inhibited by 5 mM ASA. The HCE proliferation exposed to vehicle, ANs, ANs with DNase I, ANs with Cytochalasin D, ANs with ASA or ANs with DXM were accessed by WST-1 test. **(A)** ANs promoted HCEs proliferation, which was inhibited by cytochalasin D, but not by DNase I. **(B)** DXM had no effect on the increase in AN-induced HCEs proliferation. **(C)** While 5 mM ASA slightly inhibited the increase in AN-induced HCE proliferation, 1 mM ASA had no effect on that response. Data represent mean ± SD of triplicate experiments, *p < 0.05.

HCE proliferation was accessed by the WST-1 assay. In order to distinguish between the effects of neutrophil phagocytosis and NETs, we inhibited neutrophil phagocytosis using cytochalasin D or degraded NETs using DNase I. We showed that ANs promoted HCE proliferation (*p* < 0.05), which was significantly inhibited by cytochalasin D (*p* < 0.05; Figure 6A). However, DNase I had no effect on this response (*p* > 0.05). This indicates that ANs may promote HCE proliferation via phagocytosis but not via NETs generation. Moreover, 5 mM ASA slightly inhibited the increase in AN-induced HCE proliferation (*p* < 0.05), while DXM and 1 mM ASA had no effect (*p* > 0.05; Figures 6B,C).

### ANs Inhibit HCE Migration Through NETs Formation, Which Could Be Partially Rescued by 5 mM ASA

**Figure 7:**
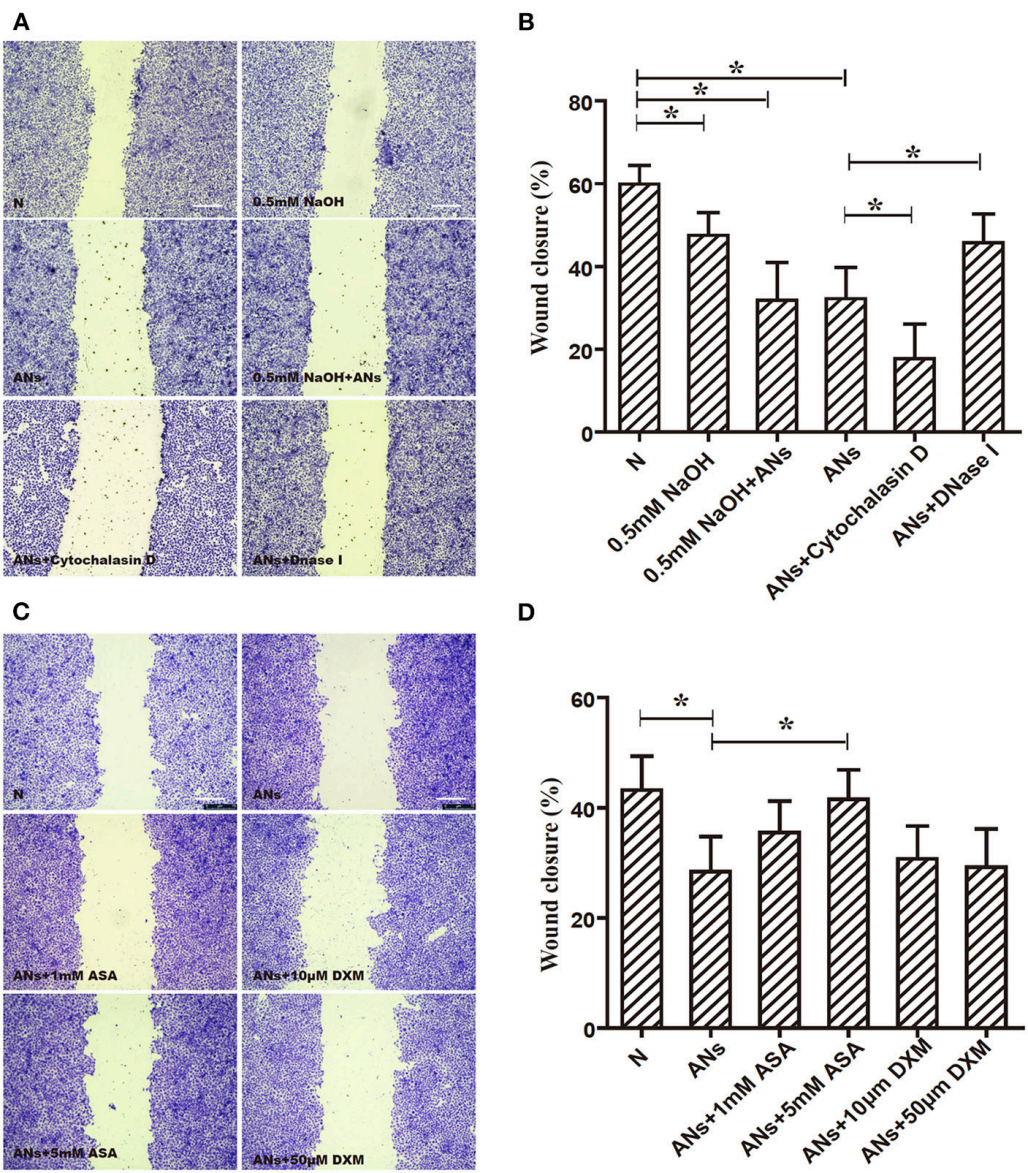
ANs inhibit HCE migration through NETs formation, which could be partially rescued by 5 mM ASA. **(A)** The HCE migration exposed to vehicle, 0.5 mM NaOH, 0.5 mM NaOH with ANs, ANs, ANs with DNase I, or ANs with Cytochalasin D were accessed by making a scratch on confluent monolayer of HCEs. **(B)** Quantification showed that 0.5 mM NaOH together with ANs significantly inhibited HCEs migration, this response was not eliminated by neutralizing the alkali in the medium with hydrochloric acid. Meanwhile, DNase I rescued AN-inhibited HCE migration (*p* < 0.05), but cytochalasin D exacerbated it. **(C)** The HCEs migration exposed to vehicle, ANs, ANs with ASA, or ANs with DXM were accessed by making a scratch on confluent monolayer of HCEs. **(D)** Quantification showed that while 5 mM ASA partially rescued AN-inhibited HCE migration, but neither DXM nor 1 mM ASA had any effect on that response. Data represent mean ± SD of triplicate experiments, *p < 0.05. Bar: 75 μm.

As shown in Figure 7A, 0.5 mM NaOH slightly inhibited HCE migration, as shown by the scratch-wound healing assay (*p* < 0.05). Moreover, although 0.5 mM NaOH together with ANs significantly inhibited HCE migration (*p* < 0.05), this response was not eliminated by neutralizing the alkali in the medium with hydrochloric acid (*p* > 0.05). Meanwhile, DNase I rescued AN-inhibited HCE migration (*p* < 0.05), but cytochalasin D exacerbated it (*p* < 0.05). These results indicate that ANs may inhibit HCE migration through the formation of NETs. Furthermore, 5 mM ASA partially rescued AN-inhibited HCE migration (*p* < 0.05). However, both DXM and 1 mM ASA had no effect (*p* >0.05; Figure 7B).

## Discussion

It is notable that the unrelenting and dysregulated corneal inflammation induced by alkali burns can lead to persistent visual impairment (20, 21). Although surgical procedures during the chronic phase may slightly improve the prognosis, visual outcomes from alkali burns remain poor (22), mainly because of insufficient control of inflammatory and proteolytic responses in tissues subjected to alkali insults. In general, neutrophils are believed to be the predominant cells in the early phase of alkali burns; they have been demonstrated to be present in the rat cornea as early as 6 h post-injury (23).

NETs formation is a newly discovered effector mechanism of neutrophils. Excessive NETs generation is known to be involved in the pathogenesis of various disorders, including autoimmune diseases, dysfunctional wound healing, and sterile inflammation (24, 25). Lately, the link between NETs and inflammatory injury after alkali burns has received more attention. Not surprisingly, we have demonstrated that NaOH could induce the formation of NETs *in vitro*. This implies that neutrophils release NETs in the injured corneal tissue after an alkali burn, which may lead to inflammatory injury and, subsequently, slower re-epithelialization, persistent ulceration, and corneal perforation. Actually, the effect of pH on NET formation has been examined before. Palaniyar and his team previously reported that alkaline pH regulates NOX-independent NET formation by promoting intracellular calcium influx, mROS generation, PAD4-mediated CitH3 formation, histone 4 cleavage, and eventually NET formation, which is often related to sterile inflammation (26). The same team also found that Nox-dependent NET formation is promoted by alkaline pH (27). Similarly, Behnen et al. noted that PMA-induced NET formation was suppressed by extracellular and intracellular acidification (28). Besides, Maueroder et al. not only found that alkaline pH promoted NET formation when induced by PMA, ionomycin, and LPS but also that an acidic pH was suppressive (29).

ASA and DXM are the two main clinically used anti-inflammatory drugs, and their effects on the extracellular-acting form of neutrophils have attracted significant attention recently. Vargas et al. reported that NETs generation in the lungs was inhibited by glucocorticosteroids in an asthma model (30). We also previously showed that DXM decreased NETs formation via a toll-like receptor (TLR)-dependent mechanism (17). Moreover, we found that glucocorticoids reduced neutrophil infiltration and inhibited NETs generation in fungal keratitis (31). However, DXM did not have a significant effect on NaOH-induced NETs generation in the present study. This divergence may be due to the different stimuli used in these studies. It has been shown that NETosis is a complex process, and that the mechanisms of NETs formation differ depending on the stimulus (32). With regards to the relationship between ASA and NETs formation, it has been reported that ASA selectively inhibits platelet-mediated NETs generation (33). Caudrillier et al. also reported that ASA treatment, through the inhibition of thromboxane (TX) A2 signaling, decreased NETs formation and subsequent transfusion-associated acute lung injury (10). In the present study, we demonstrated that ASA inhibited alkali-induced NETs formation. Similarly, Lapponi et al. results showed that ASA treatment inhibited NETs generation induced by low pH, while DXM had no effect (19).

NETs formation has been previously reported to be ROS-dependent or -independent. In the present study, we demonstrated that ROS was involved in the process of alkali-induced NETs generation. However, contrary to our prediction, there was no change in ROS levels during ASA-regulated NETs formation induced by NaOH. This suggests that ROS signaling pathways are not involved in ASA-regulated NETs formation and that ASA may modulate ROS-independent NETosis. Furthermore, we showed that NF-κB was activated during alkali-induced NETs generation. Moreover, NF-κB phosphorylation was inhibited in ASA-treated, NaOH-stimulated neutrophils, which indicates that the NF-κB signaling pathway is involved in ASA-regulated NETs generation. Similarly, previous report suggested that NF-κB activation was required for acid-induced NETs formation, and that ASA treatment inhibited NETs formation and significantly downregulated the phosphorylation of the NF-κB p65 subunit (19). Conventionally, ASA is believed to exert its anti-inflammatory functions by inhibiting cyclooxygenases (34) and subsequent prostaglandin production. Recently, Negrotto et al. demonstrated that ASA exerts anti-inflammatory actions through a novel pathway by inhibiting NF-κB-mediated survival signals and accelerating the apoptosis of neutrophils (35). Thus, ASA may prevent inflammatory responses by enhancing neutrophil apoptosis as well as inhibiting NETs formation by down-regulating NF-κB activation.

Apart from suppressing inflammation and tissue destruction, the acceleration of epithelial healing during the acute phase is another key strategy for improving the prognosis of ocular alkali burns (36). This is because the corneal epithelium is critical for preserving the integrity and function of the cornea (37). However, the role of neutrophils in epithelial healing after alkali burns is controversial. Gan et al. reported that the corneal epithelial healing rate is delayed in the absence of polymorphonuclear neutrophils (PMNs) *in vivo* (38). However, most researchers recognize the role of neutrophils in inflammatory injury. Neutrophils and their lysates have been demonstrated to significantly decelerate wound healing in rat corneal epithelial cells *in vitro* (39), and the selective suppression of neutrophils also alleviated the formation of corneal ulcerations in a porcine alkali burn model (40). Moreover, it was reported that skin wound closure was accelerated in neutrophil-depleted mice (41). Zeng et al. also noted that fasudil eye drops significantly promoted the healing of corneal epithelial defects in corneal alkali-burned mice by decreasing the infiltration of inflammatory cells (42). As neutrophils may function through distinct mechanisms, we investigated the effect of neutrophil phagocytosis and NETs formation on the proliferation and migration of corneal epithelial cells. We showed that ANs could enhance epithelial cell proliferation through the pathway of phagocytosis, which removes damaged cells and toxins from the microenvironment to allow epithelial cells to stay healthy and proliferate. Meanwhile, ANs could inhibit epithelial cell migration by generating NETs. These results may reconcile the controversial results of previous reports. In fact, it has already been demonstrated that neutrophils may exert their functions via multiple distinct pathways. On one hand, mature neutrophils promote cell regeneration and revascularization by releasing pro-angiogenic factors or growth factors from their segmented nuclei and granules upon activation (6). Conversely, NETs could be harmful to host tissues and lead to the development of various sterile inflammatory diseases (24). The role of NETs in wound healing may be further supported by a study in which NETs delayed wound healing in mice and humans with diabetes (43). Thus, we hypothesize that neutrophils may exert reparative and injurious functions through distinct pathways by promoting epithelial cell proliferation via phagocytosis and inhibiting epithelial cell migration via NETs production. To the best of our knowledge, there are no reports to date focusing on the relationship between NETs and corneal wound healing. Likewise, the role of neutrophil phagocytosis and NETs formation in corneal epithelial cell healing has not been previously described.

Currently, various strategies are being proposed to interfere with NETs formation, such as by degrading the DNA frame of NETs with DNase I or antagonizing the function of NETs-associated proteins. Previous studies have shown that preventing NETs generation could reduce the severity of tissue injuries (16, 44, 45). As a result, the regulation of NETs formation or prompt degradation of NETs may promote corneal wound healing. In the present study, we found that ASA, which inhibited alkali–induced NETs formation, had a beneficial effect on corneal epithelial cell migration. This is consistent with previous reports that non-steroidal anti-inflammatory drugs were effective in the treatment of inflammation in experimentally induced corneal alkali burns (46–48). Moreover, we found that ASA slightly inhibited the increase in AN-induced corneal epithelial cell proliferation, which is consistent with previous studies reporting that ASA inhibited proliferation and induced apoptosis in several types of human cells (49, 50). Further *in vivo* studies investigating the effects of ASA and DNase I on corneal wound healing are necessary to develop new approaches for treating corneal alkali burns.

In conclusion, we found that NETosis was highly involved in the pathogenesis of corneal alkali burns. Alkalis could induce dose- and time-dependent NETs formation, which could be inhibited by ASA, but not by DXM. ANs may interfere with HCE proliferation and migration by phagocytosis and NETs formation, respectively. ASA may enhance HCE migration by decreasing NETs formation through inhibition of NF-κB and could be a promising strategy for improving the prognosis of corneal alkali burns.

## Data Availability Statement

The raw data supporting the conclusions of this article will be made available by the authors, without undue reservation.

## Ethics Statement

The studies involving human participants were reviewed and approved by Ethics committee of Second Affiliated Hospital, School of Medicine, Zhejiang University, China. The patients/participants provided their written informed consent to participate in this study.

## Author Contributions

TW and YZ wrote the main manuscript text. YZ, TW, KY, JM, and YM performed the experiments. TW and YZ prepared the Figures 1–7. XJ designed the study and provided advice on the discussion. All authors contributed to the article and approved the submitted version.

## Conflict of Interest

The authors declare that the research was conducted in the absence of any commercial or financial relationships that could be construed as a potential conflict of interest.

## Supplementary Material

Supplementary Figure 1 Three independent Western blot results of NF-κB activation after alkali stimulation.

## References

[1] Clare G Suleman H Bunce C Dua H. Amniotic membrane transplantation for acute ocular burns. Cochrane Database Syst Rev. (2012) 9:CD009379. 10.1002/14651858.CD009379.pub2 22972141 PMC8966384

[2] Lee P Wang CC Adamis AP. Ocular neovascularization: an epidemiologic review. Surv Ophthalmol. (1998) 43:245–69. 10.1016/S0039-6257(98)00035-6 9862312

[3] Wagoner MD. Chemical injuries of the eye: current concepts in pathophysiology and therapy. Surv Ophthalmol. (1997) 41:275–313. 10.1016/S0039-6257(96)00007-0 9104767

[4] Sakimoto T Sugaya S Ishimori A Sawa M. Anti-inflammatory effect of IL-6 receptor blockade in corneal alkali burn. Exp Eye Res. (2012) 97:98–104. 10.1016/j.exer.2012.02.015 22551515

[5] Wang J. Neutrophils in tissue injury and repair. Cell Tissue Res. (2018) 371:531–9. 10.1007/s00441-017-2785-7 29383445 PMC5820392

[6] Dalli J Montero-Melendez T Norling LV Yin X Hinds C Haskard D. Heterogeneity in neutrophil microparticles reveals distinct proteome and functional properties. Mol Cell Proteomics. (2013) 12:2205–19. 10.1074/mcp.M113.028589 23660474 PMC3734580

[7] Segal AW. How neutrophils kill microbes. Annu Rev Immunol. (2005) 23:197–223. 10.1146/annurev.immunol.23.021704.115653 15771570 PMC2092448

[8] Brinkmann V Reichard U Goosmann C Fauler B Uhlemann Y Weiss DS Neutrophil extracellular traps kill bacteria. Science. (2004) 303:1532–5. 10.1126/science.1092385 15001782

[9] Jarrot PA Kaplanski G. Pathogenesis of ANCA-associated vasculitis: an update. Autoimmun Rev. (2016) 15:704–13. 10.1016/j.autrev.2016.03.007 26970490

[10] Caudrillier A Kessenbrock K Gilliss BM Nguyen JX Marques MB Monestier M Platelets induce neutrophil extracellular traps in transfusion-related acute lung injury. J Clin Invest. (2012) 122:2661–71. 10.1172/JCI61303 22684106 PMC3386815

[11] Fuchs TA Brill A Duerschmied D Schatzberg D Monestier M Myers DD Extracellular DNA traps promote thrombosis. Proc Natl Acad Sci USA. (2010) 107:15880–5. 10.1073/pnas.1005743107 20798043 PMC2936604

[12] Sonawane S Khanolkar V Namavari A Chaudhary S Gandhi S Tibrewal S. Ocular surface extracellular DNA and nuclease activity imbalance: a new paradigm for inflammation in dry eye disease. Invest Ophthalmol Vis Sci. (2012) 53:8253–63. 10.1167/iovs.12-10430 23169882 PMC3525138

[13] Barliya T Dardik R Nisgav Y Dachbash M Gaton D Kenet G. Possible involvement of NETosis in inflammatory processes in the eye: evidence from a small cohort of patients. Mol Vis. (2017) 23:922–32. 29296072 PMC5741378

[14] Lu L Reinach PS Kao WW Corneal epithelial wound healing. Exp Biol Med. (2001) 226:653–64. 10.1177/153537020222600711 11444101

[15] Ma JJ Dohlman CH Mechanisms of corneal ulceration. Ophthalmol Clin North Am. (2002) 15:27–33. 10.1016/S0896-1549(01)00017-7 12064078

[16] Manzenreiter R Kienberger F Marcos V Schilcher K Krautgartner WD Obermayer A. Ultrastructural characterization of cystic fibrosis sputum using atomic force and scanning electron microscopy. J Cyst Fibros. (2012) 11:84–92. 10.1016/j.jcf.2011.09.008 21996135

[17] Wan T Zhao Y Fan F Hu R Jin X. Dexamethasone inhibits *S. aureus*-induced neutrophil extracellular pathogen-killing mechanism, possibly through toll-like receptor regulation. Front Immunol. (2017) 8:60. 10.3389/fimmu.2017.00060 28232829 PMC5299007

[18] Stoiber W Obermayer A Steinbacher P Krautgartner WD. The role of reactive oxygen species (ROS) in the formation of extracellular traps (ETs) in humans. Biomolecules. (2015) 5:702–23. 10.3390/biom5020702 25946076 PMC4496692

[19] Lapponi MJ Carestia A Landoni VI Rivadeneyra L Etulain J Negrotto S. Regulation of neutrophil extracellular trap formation by anti-inflammatory drugs. J Pharmacol Exp Ther. (2013) 345:430–7. 10.1124/jpet.112.202879 23536315

[20] Donshik PC Berman MB Dohlman CH Gage J Rose J. Effect of topical corticosteroids on ulceration in alkali-burned corneas. Arch Ophthalmol. (1978) 96:2117–20. 10.1001/archopht.1978.03910060497024 214063

[21] Clements JL Dana R. Inflammatory corneal neovascularization: etiopathogenesis. Semin Ophthalmol. (2011) 26:235–45. 10.3109/08820538.2011.588652 21958169

[22] Luengo Gimeno F Lavigne V Gatto S Croxatto JO Correa L Gallo JL Advances in corneal stem-cell transplantation in rabbits with severe ocular alkali burns. J Cataract Refract Surg. (2007) 33:1958–65. 10.1016/j.jcrs.2007.07.020 17964405

[23] Saika S Kobata S Hashizume N Okada Y Yamanaka O. Epithelial basement membrane in alkali-burned corneas in rats. Immunohistochemical study. Cornea. (1993) 12:383–90. 10.1097/00003226-199309000-00003 8306658

[24] Jorch SK Kubes P An emerging role for neutrophil extracellular traps in noninfectious disease. Nat Med. (2017) 23:279–87. 10.1038/nm.4294 28267716

[25] Papayannopoulos V. Neutrophil extracellular traps in immunity and disease. Nat Rev Immunol. (2018) 18:134–47. 10.1038/nri.2017.105 28990587

[26] de Souza CN Breda LCD Khan MA de Almeida SR Camara NOS Sweezey N Alkaline pH promotes NADPH oxidase-independent neutrophil extracellular trap formation: a matter of mitochondrial reactive oxygen species generation and citrullination and cleavage of histone. Front Immunol. (2017) 8:1849 10.3389/fimmu.2017.01849 29375550 PMC5767187

[27] Noyan K Nguyen S Betts MR Sonnerborg A Buggert M. Human immunodeficiency virus type-1 elite controllers maintain low co-expression of inhibitory receptors on CD4+ T cells. Front Immunol. (2018) 9:19. 10.3389/fimmu.2018.00019 29403500 PMC5786543

[28] Behnen M Moller S Brozek A Klinger M Laskay T. Extracellular acidification inhibits the ros-dependent formation of neutrophil extracellular traps. Front Immunol. (2017) 8:184. 10.3389/fimmu.2017.00184 28293240 PMC5329032

[29] Maueroder C Mahajan A Paulus S Gosswein S Hahn J Kienhofer D. Menage-a-trois: the ratio of bicarbonate to CO2 and the pH regulate the capacity of neutrophils to form NETs. Front Immunol. (2016) 7:583. 10.3389/fimmu.2016.00583 28018350 PMC5145884

[30] Vargas A Boivin R Cano P Murcia Y Bazin I Lavoie JP. Neutrophil extracellular traps are downregulated by glucocorticosteroids in lungs in an equine model of asthma. Respir Res. (2017) 18:207. 10.1186/s12931-017-0689-4 29233147 PMC5727947

[31] Fan F Huang X Yuan K Zhu B Zhao Y Hu R Glucocorticoids may exacerbate fungal keratitis by increasing fungal aggressivity and inhibiting the formation of neutrophil extracellular traps. Curr Eye Res. (2020) 45:124–33. 10.1080/02713683.2019.1657464 31429304

[32] Yang H Biermann MH Brauner JM Liu Y Zhao Y Herrmann M. New insights into neutrophil extracellular traps: mechanisms of formation and role in inflammation. Front Immunol. (2016) 7:302. 10.3389/fimmu.2016.00302 27570525 PMC4981595

[33] Carestia A Kaufman T Rivadeneyra L Landoni VI Pozner RG Negrotto S. Mediators and molecular pathways involved in the regulation of neutrophil extracellular trap formation mediated by activated platelets. J Leukoc Biol. (2016) 99:153–62. 10.1189/jlb.3A0415-161R 26320263

[34] Xu XM Sansores-Garcia L Chen XM Matijevic-Aleksic N Du M Wu KK. Suppression of inducible cyclooxygenase 2 gene transcription by aspirin and sodium salicylate. Proc Natl Acad Sci USA. (1999) 96:5292–7. 10.1073/pnas.96.9.5292 10220459 PMC21857

[35] Negrotto S Malaver E Alvarez ME Pacienza N D'Atri LP Pozner RG. Aspirin and salicylate suppress polymorphonuclear apoptosis delay mediated by proinflammatory stimuli. J Pharmacol Exp Ther. (2006) 319:972–9. 10.1124/jpet.106.109389 16936242

[36] Reim M Redbrake C Schrage N. Chemical and thermal injuries of the eyes. Surgical and medical treatment based on clinical and pathophysiological findings. Arch Soc Esp Oftalmol. (2001) 76:79–124. 11228610

[37] Ueno M Lyons BL Burzenski LM Gott B Shaffer DJ Roopenian DC. Accelerated wound healing of alkali-burned corneas in MRL mice is associated with a reduced inflammatory signature. Invest Ophthalmol Vis Sci. (2005) 46:4097–106. 10.1167/iovs.05-0548 16249486

[38] Gan L Fagerholm P Kim HJ. Effect of leukocytes on corneal cellular proliferation and wound healing. Invest Ophthalmol Vis Sci. (1999) 40:575–81. 10067960

[39] Wagoner MD Kenyon KR Gipson IK Hanninen LA Seng WL. Polymorphonuclear neutrophils delay corneal epithelial wound healing *in vitro*. Invest Ophthalmol Vis Sci. (1984) 25:1217–20. 6480296

[40] Foster CS Zelt RP Mai-Phan T Kenyon KR. Immunosuppression and selective inflammatory cell depletion. Studies on a guinea pig model of corneal ulceration after ocular alkali burning. Arch Ophthalmol. (1982) 100:1820–4. 10.1001/archopht.1982.01030040800019 7138351

[41] Dovi JV He LK DiPietro LA. Accelerated wound closure in neutrophil-depleted mice. J Leukoc Biol. (2003) 73:448–55. 10.1189/jlb.0802406 12660219

[42] Zeng P Pi RB Li P Chen RX Lin LM He H. Fasudil hydrochloride, a potent ROCK inhibitor, inhibits corneal neovascularization after alkali burns in mice. Mol Vis. (2015) 21:688–98. 26120273 PMC4463969

[43] Fadini GP Menegazzo L Rigato M Scattolini V Poncina N Bruttocao A. NETosis delays diabetic wound healing in mice and humans. Diabetes. (2016) 65:1061–71. 10.2337/db15-0863 26740598

[44] Kolaczkowska E Jenne CN Surewaard BG Thanabalasuriar A Lee WY Sanz MJ. Molecular mechanisms of NET formation and degradation revealed by intravital imaging in the liver vasculature. Nat Commun. (2015) 6:6673. 10.1038/ncomms7673 25809117 PMC4389265

[45] Papayannopoulos V Staab D Zychlinsky A Neutrophil elastase enhances sputum solubilization in cystic fibrosis patients receiving DNase therapy. PLoS ONE. (2011) 6:e28526 10.1371/journal.pone.0028526 22174830 PMC3235130

[46] Laria C Alio JL Ruiz-Moreno JM. Combined non-steroidal therapy in experimental corneal injury. Ophthalmic Res. (1997) 29:145–53. 10.1159/000268009 9211467

[47] Paterson CA Pfister RR. Prostaglandin-like activity in the aqueous humor following alkali burns. Invest Ophthalmol. (1975) 14:177–83. 234925

[48] Struck HG Giessler S Giessler C. [Effect of non-steroidal anti-inflammatory drugs on inflammatory reaction. An animal experiment study]. Ophthalmologe. (1995) 92:849–53. 8563436

[49] Ma B Duan X Zhou Q Liu J Yang X Zhang D. Use of aspirin in the prevention of colorectal cancer through TIGIT-CD155 pathway. J Cell Mol Med. (2019) 23:4514–22. 10.1111/jcmm.14332 31090213 PMC6584546

[50] Pozzoli G Marei HE Althani A Boninsegna A Casalbore P Marlier L. Aspirin inhibits cancer stem cells properties and growth of glioblastoma multiforme through Rb1 pathway modulation. J Cell Physiol. (2019). 10.1002/jcp.28194 30701538

