# Supplementary figures and images for: Acetylsalicylic Acid Promotes Corneal Epithelium Migration by Regulating Neutrophil Extracellular Traps in Alkali Burn
Source: Front Immunol. 2020 Oct 15;11:551057. doi: 10.3389/fimmu.2020.551057 (PMC7593339; doi:10.3389/fimmu.2020.551057)

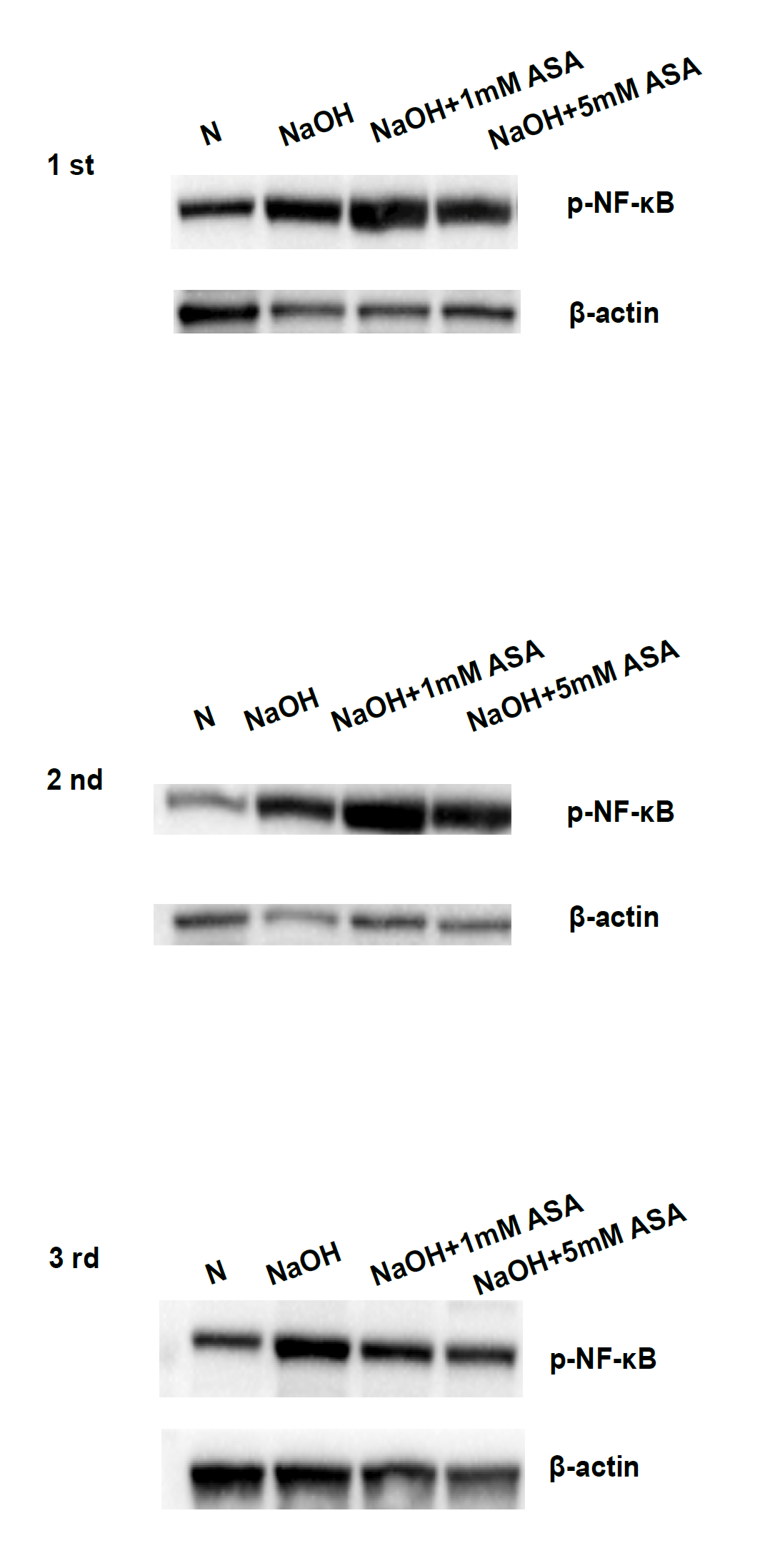

Supplement: Supplementary Figure 1 — Three independent Western blot results of NF-κB activation after alkali stimulation. [file Image_1.TIF]
